# Novel Role of the SIRT1 in Endocrine and Metabolic Diseases

**DOI:** 10.7150/ijbs.78654

**Published:** 2023-01-01

**Authors:** Chenxi Lu, Huadong Zhao, Yanqing Liu, Zhi Yang, Hairong Yao, Tong Liu, Tiantian Gou, Li Wang, Juan Zhang, Ye Tian, Yang Yang, Huan Zhang

**Affiliations:** 1 Department of Cardiology, Xi'an No.3 Hospital, The Affiliated Hospital of Northwest University. Faculty of Life Sciences and Medicine, Northwest University, Xi'an, China.; 2 Key Laboratory of Resource Biology and Biotechnology in Western China, Ministry of Education. Faculty of Life Sciences and Medicine, Northwest University, Xi'an, China.; 3 Department of General Surgery, Tangdu Hospital, The Airforce Medical University, 1 Xinsi Road, Xi'an 710038, China.

**Keywords:** SIRT1, endocrine metabolic, agonists, inhibitors

## Abstract

Silent information regulator 1 (SIRT1), a highly conserved NAD^+^-dependent deacetylase, is a cellular regulator that has received extensive attention in recent years and regarded as a sensor of cellular energy and metabolism. The accumulated evidence suggests that SIRT1 is involved in the development of endocrine and metabolic diseases. In a variety of organisms, SIRT1 regulates gene expression through the deacetylation of histone, transcription factors, and lysine residues of other modified proteins including several metabolic and endocrine signal transcription factors, thereby enhancing the therapeutic effects of endocrine and metabolic diseases. These evidences indicate that targeting SIRT1 has promising applications in the treatment of endocrine and metabolic diseases. This review focuses on the role of SIRT1 in endocrine and metabolic diseases. First, we describe the background and structure of SIRT1. Then, we outline the role of SIRT1 in endocrine and metabolic diseases such as hyperuricemia, diabetes, hypertension, hyperlipidemia, osteoporosis, and polycystic ovarian syndrome. Subsequently, the SIRT1 agonists and inhibitors in the above diseases are summarized and future research directions are proposed. Overall, the information presents here may highlight the potential of SIRT1 as a future biomarker and therapeutic target for endocrine and metabolic diseases.

## Introduction

SIRT1 is a nicotinamide adenine dinucleotide (NAD^+^)-dependent deacetylase 1, which deacetylates histone and non-histone proteins 2 and is involved in the regulation of many physiological functions, including endocrine, metabolic regulation, immune response, oxidative stress, inflammation, and ageing 3-6. As a key regulator of energy, SIRT1 affects glucose and lipid metabolism by stimulating endocrine signaling, which is associated with many molecules related to glucose/lipid metabolism, such as Adenosine 5'-monophosphate (AMP)-activated protein kinase (AMPK), Forkhead box O3 (FOXO3), Glucose Transporter 4 (GLUT4), Peroxisome proliferators-activated receptor γ (PPARγ), and Proliferator-activated receptor-gamma co-activator-1α (PGC-1α) 7-10. The activation of SIRT1 have been reported to improve insulin secretion 11. In contrast, SIRT1 deficiency leads to the decrease in the secretion of thyroid hormone 12, estrogen 13, testosterone^14^, and pituitary hormone 15, causing disorders of endocrine and metabolic system, thereby developing into obesity 16, diabetes 17, hyperuricemia 18, hyperlipidemia 19, hypertension 20. According to current research on SIRT1, this review focuses on the role of SIRT1 in endocrine and metabolic diseases. First, we sketch out the background and structure of SIRT1. Then, we highlight the relationship between SIRT1 and endocrine and metabolic diseases. Subsequently, we summarize the agonists/inhibitors of SIRT1 in endocrine and metabolic diseases. Finally, we propose future research directions between SIRT1 and endocrine and metabolic diseases. Although mature researches have been appeared on SIRT1, it is still an attractive node in the field of endocrine and metabolic diseases and provides new target for the treatment of the above diseases.

Sirtuins is also known as silent information regulator 2 (Sir2) 21, 22. Increasing the dosage or activity of Sir2 has been shown to extend the life spans of yeast, worms, and flies, while deletions or mutations of the Sir2 reverses the result 21, 23, 24. Sirtuins family consists of 7 members (SIRT1-SIRT7), which can be divided into four categories according to their structural similarity. SIRT1, SIRT2 and SIRT3 belong to type Ⅰ, SIRT4 belongs to type Ⅱ, SIRT5 belongs to type Ⅲ, as well as SIRT6 and SIRT7 belong to IVa and IVb subtypes of type IV respectively. In addition, according to their distribution, they are also divided into nuclear proteins (SIRT1, SIRT3, SIRT6 and SIRT7), plasmic proteins (SIRT2) and mitochondrial proteins (SIRT4 and SIRT5). The deacetylation activity of SIRT1 and other sirtuins need the help of NAD^+^, which is a cofactor also involved in DNA damage repair. Through deacetylation, the acetyl group of the acetylated protein substrate is transferred to the ADP-ribose (ADPR) of NAD^+^, and products such as deacetylation protein, Nicotinamide (NAM) and 2-O-acetyl-ADP-ribose 25, 26. As a classic member of Sirtuins family, SIRT1 is more conservative than other members in structure, which is of great concern 27.

**Figure 1:**
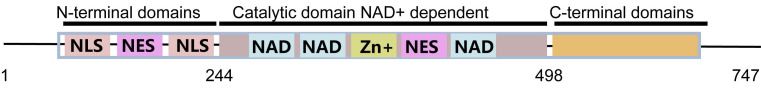
** The structure and functional domains of SIRT1 protein.** SIRT1 protein mainly contains central nuclear catalytic domain (substrate binding bag and NAD+ binding bag), nuclear localization signal (NLS) and nuclear outlet signal.

The SIRT1 gene is located on human chromosome 10 with a total length of approximately 33 Kb and contains 9 exons, 8 introns and untranslated regions 28. The human SIRT1 protein is composed of 747 amino acids and mainly contains the three structures (central nuclear catalytic structural domain, nuclear localization signals (NLS), and nuclear export signals (NES)) 4, 25, there into the catalytic structural domain covers the substrate binding pocket and the NAD^+^ binding pocket (Figure 1) 29. The histidine at position 363 of SIRT1, in which structural activity is dependent on the NAD^+^/NADH ratio in the cytoplasm, is an essential motif for deacetylation activity and involved in regulating the redox state and metabolic homeostasis 30. SIRT1 acts as an important transcriptional regulator with lysine deacetylation on histones regulating chromatin structural stability and protein activity, thus participating in maintaining normal cellular functions 31. For example, the deacetylation of SIRT1 affects the transcription of FOXO family members and PGC-1α and directly regulates lipid metabolism 32-34. SIRT1 also deacetylates lysine at position 382 of p53 protein, preventing its transcriptional activation and p53-dependent apoptosis induction 35. In addition to acetylation, other modifications of SIRT1, such as phosphorylation, ubiquitination, also partake in important physiological and pathophysiological functions. AMPK directly interacts with the deacetylase active domain of SIRT1 and phosphorylates Thr344 of SIRT1, which inhibits the deacetylation activity of SIRT1 36. Ubiquitination stabilizes proteins by covalently adding SUMO proteins to lysine residues. The Lys734of SIRT1 is modified by ubiquitin to increase the activity and stability of the protein 37. Due to its unique structure and function, SIRT1 has also been confirmed to play a positive role in other aspects of mammalian health. SIRT1 deficiency leads to energy imbalance, endocrine and metabolic disorders 38. Meanwhile, SIRT1 deficiency also promotes the occurrence of immunodeficiency, cardiovascular diseases, and aging-related diseases 39-42. Therefore, the systematic summary and review of the role of SIRT1 in endocrine and metabolism-related cells and diseases is crucial for future research.

## The role of SIRT1 in endocrine and metabolism-related cells

**Figure 2:**
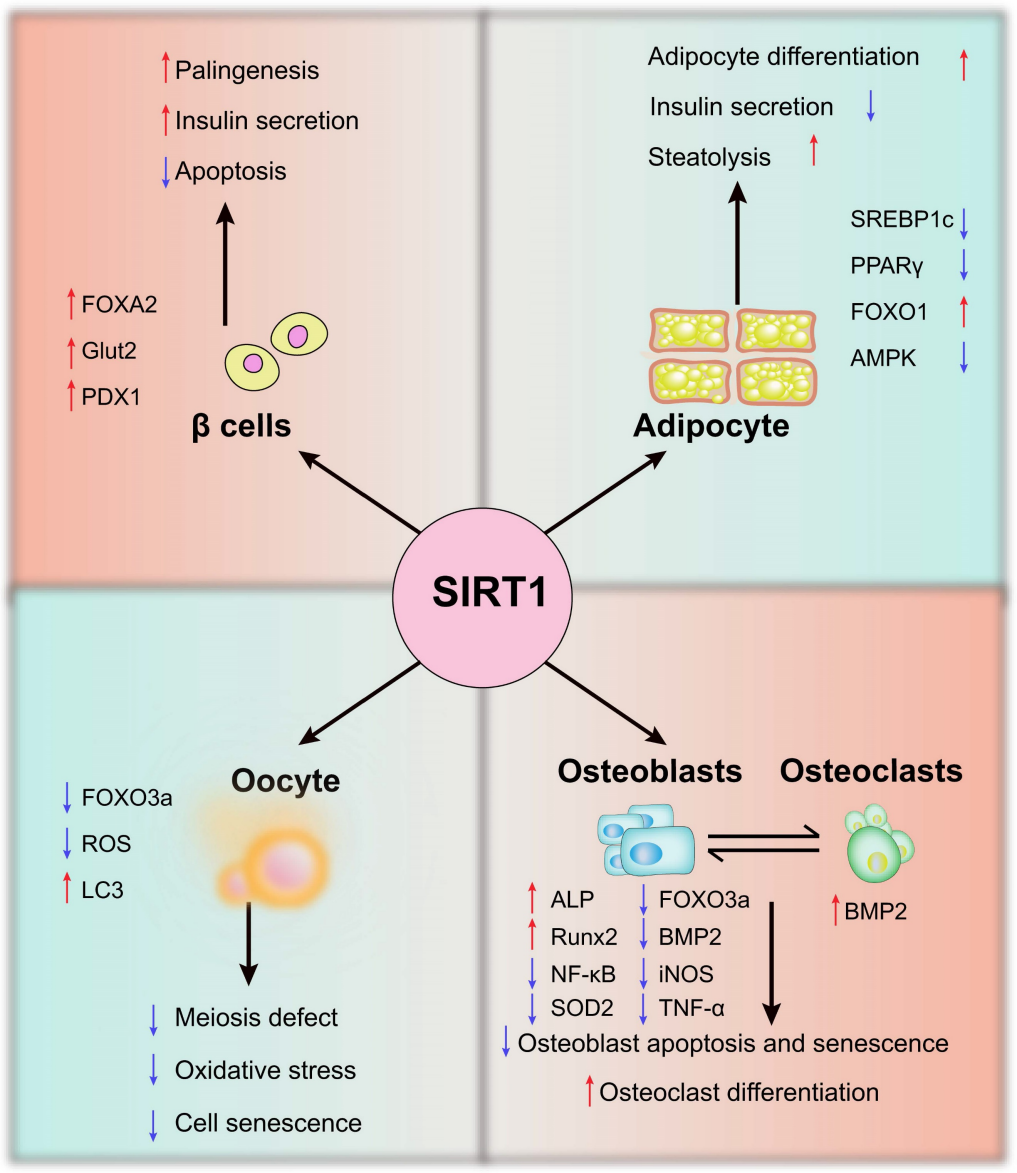
**The potential mechanisms of SIRT1 in endocrine - and metabolism-related cells.** In β cells, adipocytes, oocytes, osteoblasts, and osteoclasts, SIRT1 regulates oxidative stress, apoptosis and senescence by regulating AMPK, FOXO1, PDX1 and other molecules

Endocrine and metabolic system are interrelated and influence each other. The endocrine system consists of endocrine glands, endocrine tissues, and endocrine cells, which includes beta cells, oocytes, osteoblasts, osteoclasts, and adipocytes (Figure 2).

## Beta cells

Beta cells are insulin secreting cells in pancreatic islets, also known as B cells, accounting for about 60% of islet cells. Due to morphology and function, islet cells also contain alpha cells, delta cells, and pancreatic polypeptide cells 43. It is well known that the impairment of beta cell function leads to insulin deficiency, which increases the level of blood glucose and diabetes. In addition, pancreatic beta cell canceration promotes the generation of insulinoma, which causes symptoms of malignant hypoglycemia 44. The dynamic expression of SIRT1 was observed in endocrine progenitors both beta cell regeneration in neonatal rats and the second transition phase of mouse pancreas development. SIRT1 activation promotes beta cell regeneration by activating endocrine progenitor cells 45. Similarly, SIRT1 enhances the secretion of insulin through NAD^+^-dependent deacetylation, and counteracts inflammatory signals to avoid islet cell apoptosis 46. In addition, palmitate also decreased the expression of SIRT1 in INS-1 cells and isolated rat islets, while the result can be reversed by SIRT1. This is because SIRT1 overexpression enhances PDX1(Pancreatic and Duodenal Homeobox 1) stimulation and antagonizes FOXO1-inhibited insulin promoter activity 47. PDX1 is essential for the pancreas development and beta cells formation. Wang et al., found that pancreas-specific knockout of SIRT1 inhibits PDX1 expression and impairs islet development. SIRT1 mutant mice develop progressive hyperglycemia, glucose intolerance, and insulin insufficiency, which directly correlate with SIRT1 deletion. They further confirmed that SIRT1 interacts with and deacetylates FOXA2 on the promoter of the PDX1 gene, and positively regulates its transcription. The above results suggest that SIRT1 is involved in the regulation of beta cell formation and pancreatic development 48. Interestingly, Pinho et al., found that impairing the function of the beta cell without SIRT1 results in reduced insulin secretion, but it is not accompanied by elevated blood glucose. This may be a unique compensatory mechanism associated with decreased expression of the glucose transporter Slc2a2/Glut2 and glucagon like peptide-1 receptor as well as marked downregulation of endoplasmic reticulum 49.

## Oocytes

Endocrine system affects the development and maturity of female reproductive system and the function of female reproductive system 50. Polycystic ovary syndrome (PCOS), primary ovarian insufficiency (POI), and other diseases are frequently accompanied by oocyte damage and senescence, follicular development, and atresia dysfunction 51, 52. The ovaries are the source of oocytes and many reproductive hormones, including sex steroids, which is vital for female lifelong reproductive health. Ageing or damaged oocytes show elevated levels of reactive oxygen species (ROS) and impaired mitochondrial function, accompanied by the increased number of meiotic errors, unregulated autophagy-related proteins and early apoptosis, resulting in decreased oocyte quality and abnormal hormone secretion 53. Notably, SIRT1 regulates key transcription factors involved in aging and longevity, and is closely related to oxidative stress and autophagy 54-56. During the activation of pregranulosa cells (PGCs), oocytes and primordial follicles, the level of SIRT1 is memorably increased 57. In addition, about half of the female mice with SIRT1 deficiency in oocytes become prematurely sterile between 9-11 months of age, the reduced ability or quality of oocyte development leads to increased oxidative stress in preimplantation embryos, which inhibits cleavage divisions. In view of that, all of that originate from defiance of oocyte-SIRT1 58. On the contrary, the activation of SIRT1 not only partially prevents the deficient phenotypes of ageing oocytes, but also alleviates meiosis abnormalities and oxidative stress of oocytes 59. Autophagy degrades proteins and organelles which is degenerated and recycle their components in the cytoplasm, which is essential for the preimplantation process of early embryonic development in mammals 60. Moreover, melatonin attenuates meiotic defects in oocytes by activating SIRT1 and regulating autophagy 61. Tamura's study also confirmed that melatonin promotes the recovery of oocytes from the fallopian tube and allows normal fertilization *in vitro*. Further studies showed that the mRNA expression of SIRT1, light chain 3 (LC3), and telomere length are enhanced after melatonin treatment. Xu et al. showed that the significant increase of ROS during the postovulatory ageing inhibits SIRT1 expression, promotes the deacetylation of FOXO3a and inhibits Superoxide dismutase 2 (SOD2) expression, leading to the decrease in mitochondrial function and autophagy 62. In conclusion, these studies show that the activation of SIRT1 is essential for the development of PCOS.

## Osteoblasts and osteoclasts

Bone as an endocrine organ, which contributes to physiological regulation, cognition, glucose metabolism and hormone balance. The balance between osteoblasts and osteoclasts determines the quality of bone 63. SIRT1 expression is reduced in cartilage and subchondral bone plate in patients with osteoarthritis 64. In addition, SIRT1 maintains the balance between bone formation and resorption by regulating the ratio of osteoblasts to osteoclasts 65. A recent study also found that resveratrol (Res), the SIRT1 agonist, significantly improved bone quality and reduced serum alkaline phosphatase and osteocalcin levels in the rats with osteoporosis 66. Osteocalcin is a hormone secreted by osteoblasts and has been shown to be involved in insulin secretion, insulin resistance and energy expenditure 67. In MC3T3-E1 cells, inflammation increase apoptosis and decrease alkaline phosphatase (ALP) activity. While overexpression of SIRT1 inhibits osteoblast apoptosis, increases ALP activity and the expression of runt-related transcription factor 2 (Runx2) and osteocalcin. It was also found that overexpression of SIRT1 protects osteoblasts against tumor necrosis factor-α (TNF-α)-induced cell injury, at least in part, by repressing NF-κB activity and genes downstream of NF-κB, including iNOS. Specifically, TNF-α promotes iNOS expression and NO production by mediating NF-κB signaling pathway, while SIRT1 overexpression can reverse these results 68. Notably, Runx2 is the gene that encodes for the protein involved in the osteogenic differentiation process from mesenchymal precursors 69. Runx2 haploinsufficiency leads to the skeletal disorder characterized by bone and dental abnormalities known as cleidocranial dysplasia 70. Hong et al. found that atorvastatin increases bone mass and promotes osteogenesis in ageing apolipoprotein E-deficient mice by activating SIRT1-Runx2 axis 71. Additionally, Res increases the formation of SIRT1 and FOXO3a complex, regulates the activity of Runx2 promoter, and promotes ossification of human MSCs 72. Bone morphogenetic protein 2 (BMP2) is the key factor in inducing cartilage differentiation. SIRT1 can promote the BMP2-induced cartilage differentiation of MSCs and reduce the apoptosis and decomposition of extracellular matrix under oxidative stress 73. Zhao et al. also highlighted that Res dose-dependently increases both ALP and endothelial nitric oxide synthases (eNOS) levels, increase ALP, Runx2 and BMP2 and stimulate bone formation. On the contrary, SIRT1defiance reduces eNOS, BMP2 and ALP. The evidences describe above suggest that SIRT1 is the key molecule which regulates osteoblast differentiation and bone homeostasis 74.

## Adipocytes

Adipocytes are essential for regulating pathological conditions such as obesity, diabetes and metabolic syndrome 75. SIRT1 knockout porcine preadipocytes are reduced and apoptosis is increased 76. Additionally, the mice with adipocyte-selective deletion of SIRT1 are more susceptible to diet-induced insulin resistance, which is associated with the increase in the number of adipose-resident macrophages and their polarization to the pro-inflammatory M1 subtype 77. It has been reported that SIRT1 inhibits the transcriptional activities of PPARγ (the key factors in adipocyte differentiation) and sterol regulatory element-binding protein 1c (SREBP1c) through deacetylation, thus suppressing adipocyte differentiation, reducing fat accumulation, and promoting fat mobilization 78, 79. SIRT1 overexpression can deacetylate Lys293 and Lys268 of PAPRγ and induce white adipose tissue remodeling 80. However, in SIRT1 knockout mice, fatty acid mobilization of white adipocytes is disrupted after fasting, and SIRT1 also significantly inhibits PPARγ in adipocytes. Repression of PPAR-γ by SIRT1 is also evident in adipocytes, where overexpression of SIRT1 attenuates adipogenesis, and RNA interference of SIRT1 enhances it 79. FOXO1 can be localized to the nuclear, cytoplasmic and mitochondrial compartments of adipocytes, affecting the source of ROS 81. The level of FOXO1 is decreased in adipocytes of db/db mice. Res can transport FOXO1 to the nucleus by activating SIRT1 and increase the level of FOXO1 in adipocytes 82. In addition, SIRT1 controls acetylation status and functional activity of FOXO1 that directly binds to the adipose triglyceride lipase (ATGL) promoter and regulates the transcription of ATGL gene, reducing the expression of AMPK in adipocytes 83 Activated SIRT1 reduces phosphorylated-FOXO1 expression, thereby activating FOXO1 and inhibiting adipogenesis of adipocytes 84. Taken together, the above results indicate that SIRT1 is a novel adipocyte regulator that is associated with multiple signaling pathways such as PPARγ and FOXO1 to regulate adipocyte differentiation, fat accumulation and energy metabolism.

## The role of the SIRT1 in endocrine and metabolic diseases

Under pathological conditions, endocrine disorders lead to abnormal function of endocrine glands, and then endocrine and metabolic diseases. SIRT1 in mammals can regulate the expression of target genes through various modifications, and plays an important role in endocrine and metabolic diseases 85, 86.

## Hyperuricemia

Uric acid is a metabolite of purine, and it can increase and eventually lead to gout/hyperuricemia when there is a disorder of purine metabolism or uric acid excretion 87. Hyperuricemia is a common endocrine and metabolic disease in middle-aged and elderly men, which has seriously harmed human health 88. The traditional Chinese herb *Smilax china* L (effective component Res) has been used to treat hyperuricemia, gout and related kidney diseases. Moreover, studies have found that Res can reduce xanthine oxidase (XO), serum uric acid level, uric acid excretion fraction and blood urea nitrogen to normal state 89, 90. In the mouse with hyperuricemia, the expression of ATP-binding cassette subfamily G member 2 in ileum was activated by acetylation PGC-1α/PPARγ pathway after SIRT1 activation by Res, reducing the level of serum uric acid, thus exerting an anti-hyperuricemia effect 91. Additionally, hyperuricemia promotes the proliferation of vascular smooth muscle cells (VSMCs) via activating the renin-angiotensin-aldosterone system, causing renal vasoconstriction and glomerular arterial wall thickening 92, 93. Ma et al. found that Simiao pill restores high fructose-induced hyperuricemia and metabolic syndrome by up-regulating SIRT1 in glomerular of mice with high-fructose, inhibiting NF-κB pathway and the activation of NOD-like receptor pyrin domain containing 3 (NLRP3) inflammasome, improving interstitial infiltration of nephritis cells and glomerular injury and reducing urinary albumin level 94. Polydatin has also been shown to inhibit NF-κB/NLRP3 through the AMPK/SIRT1 pathway, thereby reducing potassium oxonate-induced hyperuricemia and renal inflammation 18. Xu et al. also found that hyperuricemia was associated with decreased SIRT1, phosphorylation of downstream target molecules FOXO3a, increased expression of androgen receptor, XO and deacetylation of NF-κB subunit p65. Importantly, they further found that hyperuricemia is more frequent and widespread in men with nonalcoholic fatty liver disease (NAFLD) than in women, which is intimately contributed to the inhibition of SIRT1 signaling pathway induced by hyperuricemia 95, 96. Interestingly, elevated estrogen levels caused by SIRT1 may also be responsible for treatment of hyperuricemia. H Sumino pointed out that postmenopausal women with hyperuricemia are treated with hormone replacement therapy to reduce serum uric acid level 97, 98. In conclusion, these studies suggest that SIRT1 may be a potential target of hyperuricemia and its complications, which is of great significance for further clinical research and application.

## Hypertension

Hypertension is a major risk factor for premature death and disability worldwide 99, 100. The pathogenesis of hypertension can also lead to dysfunction of the nervous system 101, endocrine system 102 and immune system 103. The information connection between these systems is mainly accomplished by neuropeptides and endocrine hormones, including neuropeptide Y 104, angiotensin II (Ang II) 105, arginine-vasopressin 106, endothelin 107 and NO 108. Ryohei et al. reported that Res suppresses the expression of AT1R in the mouse aorta by activating SIRT1 and ameliorates Ang II-induced hypertension. Meanwhile, overexpression of SIRT1 reduces the expression of AT1R, while SIRT1 inhibitor (nicotinamide) reverses this effect. Further studies showed that the suppression of AT1R depends on the most proximal promoter region, which contains the Sp1 binding site (GC box). GC box mutation of luciferase construct failed to respond to Res, suggesting that Sp1 site plays an important role in Res-induced AT1R downregulation 109. Nicotinamide phosphoribosyl transferase (NAMPT) is a potential cardiovascular protective effect of adipose cytokines, which plays an important role in DNA damage repair and prevent premature VSMCs in aging, and is also a key enzyme regulating NAD^+^ biosynthesis and SIRT1 activity 110, 111. The overexpression of NAMPT partially inhibits Ang II-induced elevated ROS levels by regulating SIRT1 and the concentration of NAD^+^, thus relieving Ang II-induced hypertension. This observation suggests that NAMPT may regulate the occurrence of hypertension through SIRT1 112. In addition, endothelial dysfunction is also considered as a possible early key link in the occurrence of hypertension 113. Extracellular vesicles collected from induced pluripotent stem cell-derived mesenchymal stem cells, may reduce age-related endothelial dysfunction, arteriosclerosis, and hypertension by activating the SIRT1-AMPKα-eNOS pathway 114. Grape seed proanthocyanidin extracts (GSPE) indirectly up-regulates SIRT1 and inhibits aortic NO production disorder, improving hypertension and showing the potential of anti-inflammatory, antioxidant, anti-ageing and regulation of endothelial function 115-117. Klotho, an ageing-suppressor gene, whose mutations results in significant increases in pulse wave velocity and blood pressure. Importantly, Gao et al., pointed out that serum Klotho deficiency in hypertensive patients is associated with significantly reduced activity of AMPKα, SIRT1, and eNOS in aortic endothelial cells (ECs), along with collagen expression and elastin breakdown. Conversely, activation of SIRT1 functionally interacts with AMPKα, up-regulates phosphorylation of AMPKα and then activates eNOS, inhibits ROS accumulation and oxidative stress in aortic ECs and leads to vascular remodeling, down-regulates collagen expression and elastin breakage, predicting the improvement of aortic sclerosis and hypertension 118.

## Polycystic ovarian syndrome

PCOS is the most common endocrine and metabolic disordered disease in the women 119, 120. This may be the result of hypothalamo-pituitary ovarian axis disorder, follicular membrane cells or granulosa cells (GC) dysfunction, and metabolic abnormalities 121-123. PCOS is associated with specific reproductive health complications, including lower oocyte quality and clinical pregnancy rates in assisted conception cycles. Metformin, an anti-aging agent, is approved for the treatment of PCOS 124. Clinical trials and observational studies have found that metformin can prevent or mitigate PCOS through SIRT1-related pathways 125. Increased testosterone level is the pathological feature of PCOS patients, and when the PCOS patients are treated with Res, the level of serum testosterone decreases, the number of secondary and closed follicles increases, while Graafian follicles decrease. It was further found that the combination of metformin and Res induce the antioxidant and anti-inflammatory systems of PCOS by activating SIRT1 and AMPK, thereby improving the weight gain, hormone levels and follicular cell structure of PCOS 126. Tao et al. found that SIRT1 expression in PCOS group is significantly lower than that in the control group in the establishment of PCOS rat induced by dehydroepiandrosterone, and shows the loss of estrous cycle, saccular dilatation of the follicle, reduce luteal number, and thickens follicular membrane cell layer 127. The researcher also found that both metformin and exenatide improve reproductive endocrine function in PCOS rats via the AMPKα-SIRT1 pathway 128. It is suggested that the deficiency of SIRT1 and estrogen promotes the occurrence of diseases related to female reproductive development. In addition, quercetin can up-regulate the expressions of AMPK and SIRT1 in ovarian tissues, and reverse the changes of adiponectin, visfatin and resistin in adipose tissues of PCOS rats, thus maintaining hormone and metabolic balance 129. Interestingly, SIRT1 knockdown in human ovarian GC inhibits estrogen synthesis activity and aromatase 130. Previous studies have pointed out that SIRT1 regulates non-histone activity and affects aromatase transcription regulation 131. However, enhanced aromatase activity will increase PCOS susceptibility 52. SIRT1 plays an important role in the genesis and development of PCOS, which provides a basis for the development of potential therapeutic methods to improve the metabolism and reproductive function of PCOS.

## Osteoporosis

Osteoporosis is the disease induced by genetic and environmental interference with the endocrine system 132. With aging, the decrease of NAD^+^ leads to the decrease in bone progenitor cells and bone mass, accompanied by the imbalance in the number and activity of osteoblasts and osteoclasts 133, 134. Menopausal, ovariectomized female mice and aged male mice exhibit decreased SIRT1, osteoporosis and bone injury 135. Meanwhile, SIRT1 knockout mice, as well as osteoblast and osteoclast specific knockout, show a low bone mass phenotype 136. Significantly reduced bone mass, reduced bone formation and increased bone marrow adipose formation are observed in female SIRT1 haplo-insufficient (Sirt1^+/-^) mice, along with osteoarthritis 137, 138. Importantly, clinical studies have identified the potential of SIRT1 in predicting and treating related diseases such as osteoporosis and osteonecrosis. SIRT1 expression in femoral neck was significantly reduced in patients with osteoporosis 139. Additionally, in 16 female patients with osteoporosis, there is a negative correlation between SIRT1 activity in peripheral blood mononuclear cells and C-terminal cross-linking telopeptide of type I collagen, the marker of bone resorption in serum 140.

Besides, overexpression of SIRT1 inhibits H_2_O_2_-induced osteoblast apoptosis by activating the FOXO1/β-catenin pathway 141. One estrogen, 17β-E2 (10^-6^ M) up-regulates SIRT1, p-AMPK and FOXO3a in osteoblasts, thereby inhibiting osteoblast apoptosis by promoting autophagy 142, while the protective effect of autophagy may be attributed to reducing intracellular oxidative damage and maintaining cell structure and function 143. Res and endoplasmic reticulum stress (ERS) inhibitor 4-PBA significantly inhibit osteoclast differentiation and osteolysis 144. Activation of SIRT1 can regulate the activity of osteoclasts and osteoblasts and improve bone metabolism, thereby reducing osteoporosis 145. In conclusion, SIRT1 plays a positive role in maintaining bone homeostasis, bone mineralization and bone resorption, which also brings hope for the treatment of osteoporosis.

## Diabetes

As a metabolic disease, diabetes is characterized by defective insulin secretion or insulin dysfunction leading to impaired islet beta cell function 146. Study confirmed that the SIRT1 level was always lower in patients with poor glycaemic control than in those with good glycaemic control 147. In the isolated rat islets, SIRT1-mediated NF-κB deacetylation inhibits iNOS and cytokine-mediated beta cell damage 148. SIRT1 activation also promotes beta cell recovery and endocrine progenitor differentiation 45. These studies revealed that SIRT1 plays an active role in regulating insulin secretion. Notably, direct sequencing and exon sequencing of a patient with type 1 diabetes revealed t-to-C exchange in SIRT1 exon 1 and excessive production of nitric oxide, cytokines and chemokines, suggesting that SIRT1 mutation may be a potential weakness of patients with diabetes 149. There are many strategies and drugs for the treatment of diabetes in clinic, among which metformin is the star of clinical drug. Hyperglycemia induced expression of inflammatory genes, NF-κB and the proapoptotic gene Bax in bovine retinal capillary endothelial cells (BRECs) and diabetic rat's retinas. BRECs with knockdown SIRT1 increases sensitivity to hyperglycemic stress, while SIRT1 overexpression or metformin activation inhibit mitochondrial ROS-mediated PARP activity and glyceraldehyde-3-phosphate dehydrogenase through upregulation of LKB1/AMPK, and ultimately inhibiting NF-κB and Bax expression. It was proved that metformin may be associated with the SIRT1/LKB1/AMPK pathway in inhibiting diabetic retinopathy 150. High glucose treatment significantly reduces the expression of SIRT1 protein in mouse microvascular ECs. However, overexpression of SIRT1 or metformin can attenuate the decreased expression of SIRT1 induced by high glucose, thus regulating downstream targets FOXO1 and p53/p21, and protecting ECs from high glucose-induced premature aging 151. In addition, SIRT1 is also involved in the occurrence of other diabetes-related diseases. Decreased hepatic glucose production in type 2 diabetes rats with reduced hepatic SIRT1 levels, and systemic SIRT1 activation induced by drugs or genes can prevent dietary diabetes 152. Knockout of SIRT1 leads to hyperglycemia and insulin resistance in the liver 153. Importantly, the positive effect of SIRT1 has also been confirmed in diabetic nephropathy 154, 155 and diabetic cardiomyopathy 156.

## Hyperlipidemia

Hyperlipidemia is a common disease of dyslipidemia caused by endocrine and metabolic disorders 157, 158. Hyperlipidemia is clinically divided into the following categories: hypercholesterolemia, hypertriglyceridemia, mixed hyperlipidemia and atherosclerotic dyslipidemia 159. SIRT1 is the major regulatory factor of lipid and carbohydrate metabolism. The reduction of SIRT1 will cause metabolic disorders, fatty liver and obesity 160. Reciprocally, enhancing the activity of SIRT1 may normalize abnormal fat morphology and abnormal expression of lipid metabolism markers, thus regulating cholesterol and lipid metabolism 161, 162. SIRT1 is involved in the caspase-1 pathway in early hyperlipidemia and promotes ECs activation prior to monocyte recruitment 163. In addition, SIRT1 is closely associated with lipid metabolism markers, including PPARα/γ 80, SREBP 164, Liver X Receptor α (LXRα) 165 and low-density lipoprotein (LDL) receptor 166. For example, the accumulation of oxidized LDL (oxLDL) in peritoneal macrophages of SIRT1-deficient mice increases and promotes the formation of foam cells. SIRT1 reduces the expression of lectin-like oxLDL receptor-1 by inhibiting NF-κB signaling pathway, thus reducing oxLDL uptake and alleviating atherosclerosis 167. SIRT1 also leads to lipolysis of mature adipocytes by enhancing the activity of PPARα 79 and directly deacetylates SREBP during fasting, which leads to inhibition of lipid synthesis and fat storage 164. In addition, PPARα may indirectly affect lipid synthesis through cross-talk with SREBP and exploit the advantages to the full in regulating cellular fatty acid and cholesterol homeostasis 168, 169. AMPK is a nutrition-sensing molecule which correspondingly reduces fatty acid synthesis 170. Increased SIRT1 and AMPK activity inhibits dysregulation of lipids and obese phenotypes 171. In the hyperlipidemia-induced hepatic steatosis and atherosclerotic mice, SIRT1 restores cholesterol efflux caused by hyperlipidemia through regulating the LXRα/β-PPARγ pathway 172. Notably, there are several compounds which alleviates lipid metabolism diseases such as hyperlipidemia by activating SIRT1. As the cofactor of SIRT1, nicotinic acid increases p-AMPK and SIRT1 in adipocytes and myotube, reduces total cholesterol, cholesterol esters, plasma triglycerides, and lessens the size of atherosclerotic lesions and lipid area 173. Ginsenoside Rb2 prevents hepatic lipid accumulation *in vivo* and *in vitro* through SIRT1-mediated autophagy induction 174. Melatonin improves serum biochemical markers and liver morphological damage, and inhibits oxidative stress through its antioxidant properties and upregulation of SIRT1 175. Importantly, He et al. assessed the association between serum SIRT1 levels and coronary atherosclerotic plaque characteristics by computed tomography angiography (CTA) and Framingham Risk Score generation in each patient. The results showed that serum SIRT1 level is significantly reduced in the non-high-risk plaque group. It is suggested that SIRT1 may play a predictive role in coronary artery pre-CTA plaque screening 176. These evidences confirm the positive effect of SIRT1 activation on hyperlipidemia.

**Figure 3:**
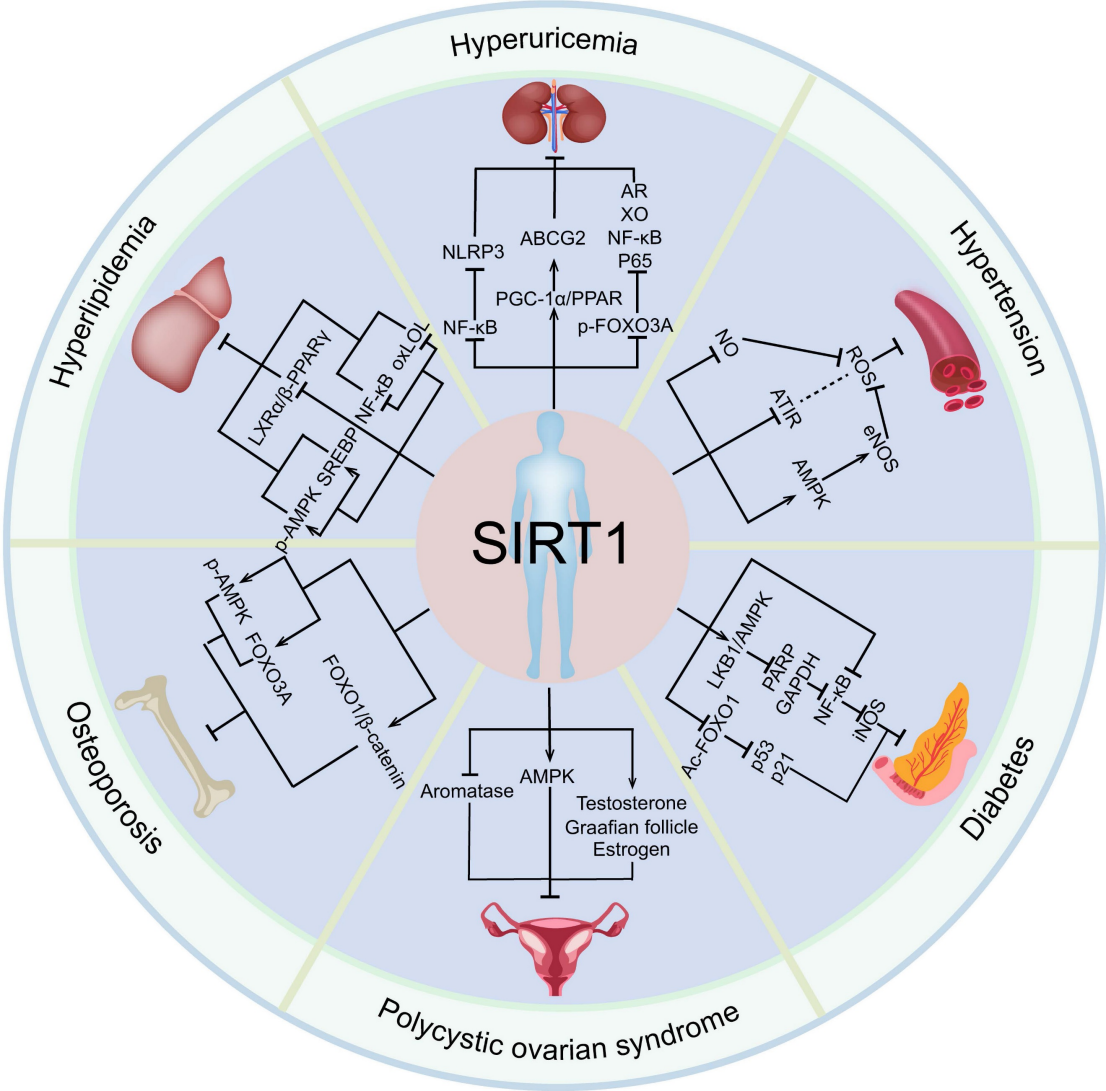
** The relationship between SIRT1 and endocrine and metabolic diseases.** Activated SIRT1 can prevent the occurrence and development of various endocrine and metabolic diseases, including hyperuricemia, hypertension, polycystic ovary syndrome, osteoporosis, diabetes, and hyperlipidemia.

In conclusion, SIRT1 has a significant protective effect on a variety of endocrine diseases, such as hyperuricemia, hypertension, polycystic ovarian syndrome, osteoporosis, by regulating a variety of target genes (Figure 3). Importantly, SIRT1 have been widely studied in clinical practice and gradually become key molecules in the treatment of endocrine and metabolic diseases and aging related diseases 177.

## Application of SIRT1 agonists in endocrine and metabolic diseases

A large number of studies have shown that SIRT1 has positive effects on a variety of diseases, along with the development of different SIRT1 activators, including Res 178, SRT2183 179, SRT1460 180, SRT1720 181, SRT2104 182 and SRT3025 183 (Table 1).

## Res

Res is a natural polyphenol with strong biological activity, also known as astragalus triphenol 184. Res has been paid attention to by the medical community, with anti-oxidation, antibacterial, anti-inflammatory, anti-aging and estrogen-like activities 185-187. Many formulations containing Res have been shown to be beneficial in healthy, obese male by reducing lipid content, circulating glucose, triglycerides and inflammatory markers in the liver 188-190. The combination of Res with SIRT1 promotes the conformational change of SIRT1 and enhances its activity 191. Res affects thyroid function by enhancing iodide ion capture, and increases thyrotropin secretion by activating SIRT1^192^. It also enhances insulin sensitivity and reduces hepatic glucose production 17. In addition, Res promotes browning in a SIRT1-dependent manner and has a beneficial effect on excess fat utilization, suggesting potential therapeutic application of Res in the treatment of obesity and related metabolic disorders 54. Sara et al. systematically analyzed the effects of Res in animal and clinical studies. Many animal studies have reported beneficial effects of Res on sex hormones, gonadotropins, and blood glucose. In particular, Res improves ovarian volume, high-quality oocyte rate, high-quality embryo rate, androgen, and gonadotropin concentration in PCOS patients 193. A meta-analysis was conducted to review the effects of Res intake on weight loss. This study showed that the supplementation of Res significantly reduces body weight, body mass index, waist circumference, and fat mass, and increases lean mass 194. It is worth noting that a clinical study assessed the effects of short-term high-dose Res administration on intestinal and hepatic lipoprotein turnover and insulin sensitivity in non-diabetic, overweight, and obese male who had mild hypertriglyceridemia. Res was given at doses of 1000 mg/day for one week and increased to 2000 mg/day the second week. In this study, Res improved glucose tolerance without causing any adverse reactions, however, there was no significant effect on insulin sensitivity and plasma triglyceride content, which may be related to the time and concentration of the drug 190. Notably, Res has a positive effect on metabolism (URL: www.clinicaltrials.gov. Unique Identifier: NCT01451918.) 190. Interestingly, Res reduced cerebrospinal fluid (CSF) metalloproteinase 9 (MMP9), modulated neuroinflammation, improved mild-to-moderate Alzheimer's disease, and induced adaptive immunity. SIRT1 activation may be a viable target for the treatment or prevention of neurodegenerative diseases 177. Additionally, in vivo res pretreatment confers neuroprotection similar to ischemic preconditioning (IPC) via the SIRT1-UCP2 (mitochondrial uncoupling protein 2) pathway 195. Furthermore, potential effect modifications by sex, smoking and vascular risk factors of the SIRT/UCP genes in the associations with atherosclerotic plaque 195. Meanwhile, polymorphisms in SIRT6/UCP1 genes may be important for increased carotid plaque burden and echodensity, but translation of these findings to an individual risk of cerebrovascular events needs further investigation 196. These basic studies and randomized controlled trials provide potential evidence for the clinical application and development of Res as a daily dietary supplement.

## SRT3025

As the SIRT1 small molecule activator with oral activity, SRT3025 increases the expression of hepatic LDL receptors and accumulation of proprotein convertase subtilisin/kexin type 9 (Pcsk9), reduces plasma cholesterol level, inhibits inflammatory response and atherosclerosis 183. Estrogen deficiency can lead to rapid bone loss and skeletal fragility. Oral administration of SRT3025 (50 and 100 mg/kg/d) for 6 weeks completely reverses the harmful effects of ovariectomy on bone mass and bone structure. SRT3025 achieves its therapeutic effect by decreasing the expression of bone sclerostin, increasing cortical periosteal mineralizing surface and serum propeptide of type I procollagen (a bone formation marker). Additionally, SIRT1 inhibitor EX-527 reverses the positive effects of SRT3025 *in vitro*. This study provides a theoretical basis for SRT3025 in metabolic and age-related diseases such as osteoporosis 197. Diabetic mice treated with SRT3025 has significantly improved blood glucose, reduced islet alpha cell mass and decreased plasma glucagon concentration. Consistent with the decrease in glucagon abundance, overexpression of key gluconeogenic enzymes, glucose-6-phosphatase and phosphoenolpyruvate carboxykinase (PCK1), which are associated with diabetes, are also decreased by SRT3025 198. In addition, pharmacological activation of SIRT1 by SRT3025 increases the expression of several thermogenic genes FOXC2, PGC-1α, Dio2, TFAM and Cyc1 in C3HT101/2 cells. Notably, SRT3025 treatment increases PGC-1α mRNA and protein levels through activating SIRT1 in femoral MSCs in female patients undergoing hip operations caused by fracture or osteoarthritis. These evidences confirm that SRT3025 activates SIRT1 and upregulates PGC-1α to stimulate a thermogenic gene program in mouse and human bone marrow adipocytes 199. SRT3025 inhibits the expression of sclerotin in osteocytes and thus inhibits age-related bone loss 200. Importantly, a phase I clinical trial of SRT3025 at different doses in the treatment of diabetes has been completed (NCT01340911). This evidence will provide outstanding guidance for the development and application of SIRT1 agonists.

## SRT2183

SRT2183, a selective SIRT1 activator, can bind to SIRT1 enzym-peptide substrate complex, reduces the Michaelis constant for acetylated substrates, and directly activates SIRT1 through allosteric mechanism 181, 201. The EC_1.5_ (the compound concentration required to increase enzyme activity by 50%) of SRT2183 is about 0.36 μM, and the maximum activation is 296%. SRT2183 reduces the acetylation of SIRT1 substrate p53. SRT2183 is used as a positive control in SIRT1 deacetylation because of its good tolerability 181. SRT2183 activates the expression of AMPK and SIRT1 and reduces the acetylation level of lysine 310 of RelA/p65^202^. In bone marrow macrophages, SRT2183 inhibits RANKL-induced osteoclast formation and resorption ability, suggesting that SRT2183 plays a positive role in bone metabolism 202. Moreover, high glucose exposure induced the expression of p53, SIRT1 and AMPK in HepG2 cells. SRT2183 reverses this result and reduces triglyceride accumulation and cytoplasmic oxidative stress 203. In addition, many metabolic syndromes are associated with reduced kidney function, and the medulla is critical in regulating water and sodium balance and maintaining normal blood pressure. Activated SIRT1 by SRT2183 reduces apoptosis and increases fibrosis in unilateral ureteral obstruction 204. Recent studies have shown that SRT2183 also inhibits the growth of ovarian cancer cells. In terms of mechanism, SRT2183 has anti-ovarian cancer effects by activating the apoptosis pathway and increasing the level of LC3II, enhancing the degradation of p62/SQSTM1, and inducing the maturation of autophagosomes 205. However, compared with other SIRT1 agonists, there are few studies on SRT2183 at the present stage, especially in animal experiments. Further studies are needed to explore SIRT1-related agonist differences and feedback regulation in endocrine and metabolic pathways.

## CAY10602

VASANTHA et al. performed a high-throughput screening of 147,000 compounds. Compounds with relative percentages greater than or equal to 150 are considered definitive SIRT1 activators. CAY10602 increases SIRT1 activation and significantly inhibits TNF-α expression 206. As a cytokine generated by macrophages, TNF-α is involved in adipose tissue metabolism and endocrine function 207, 208. High-fat-diet increases body weight, serum total cholesterol, triglycerides, aspartate aminotransferase, alanine aminotransferase, blood glucose, insulin levels, and liver malondialdehyde, while decreases liver superoxide dismutase activity. These changes are negatively correlated with SIRT1 and PGC-1α. HepG2 hepatocytes cell line exposed to oleic acid (OA) for 48 h shows decreased cell viability, apoptosis, lipid accumulation and ROS production, while pretreatment with CAY10602 at 20μM for 2h reverses this effect. On the contrary, pretreatment with Tenovin-6 aggravates the effect of OA on hepG2^209^. CAY10602 restores phosphorylation of eNOS (p-eNOS), p-AMPK, and phosphorylation of Akt (p-Akt) levels inhibited by high glucose in diabetic mice. These results suggest that CAY10602 contributes to the beneficial effects of SIRT1 on endothelial function in diabetes and obesity 210.

## Others

In addition to Res, SRT3025, CAY10602, and SRT2183, there are many other small molecular compounds that can activate SIRT1, including SRT1460, SRT1720, and SRT2104. The EC1.5 of SRT1460 is 2.9μM and the maximum activation rate is 447%. The dissociation constant and reaction enthalpy of SRT1460 confirm that SRT1460 binds to a SIRT1-peptide substrate complex and promotes a more productive conformation that improves catalytic activity 181. In addition, SRT1720 is also a small molecule activator of SIRT1 with an EC1.5 of 0.16 μM and a maximum activation value of 781%. SRT1720 has a clear protective effect on diabetic nephropathy, which originates from that SRT1720 inhibits the expression of HIF1α, GLUT1 and SNAIL, thereby reducing glomerular hypertrophy, mesangial expansion, glomerulosclerosis, and interstitial fibrosis 211. SRT1720 also up-regulates the expressions of SIRT1, SIRT6, FOXO3a and NRF-1, inhibits the expressions of mTORC1, p-MTOR, p-P70S6K, NF-κB and p53, improves the follicular reserve of diet-induced obese female mice, and prolongs the ovarian life 212. SRT2104 inhibits dysfunction in ECs treated with high glucose 213. It is worth noting that natural products also have a positive effect on regulating endocrine and metabolic diseases. For example, naringenin and hesperetin help to improve impaired thyroid function in the old-aged rats 214. Isoflavonoid can affect the expression of SIRT1 and regulate the electrophysiology of hypothalamic neurons related to the secretion of gonadotropin-releasing hormone (GnRH), controlling hormone release and reproductive maturation 215. In addition, vitamin D 216, melatonin 217, natural carotene 218, berberine 219, ferulic acid 220 and other natural products affect the expression of SIRT1 to varying degrees, and then participate in the regulation of endocrine and metabolic systems.

## Application of SIRT1 inhibitors EX527 in endocrine and metabolic diseases

In addition, inhibitors of SIRT1 have also been widely studied, including EX527 (Selisistat)^221^, Sirtinol 222, Inauhzin 223, SIRT1/2 Inhibitor IV 224. EX527 has been widely studied and applied in a variety of diseases. For example, In DM mice, EX527 inhibited promyelocytic zinc finger protein (PLZF) and insulin induced by Far-infrared (FIR) radiation, respectively. SIRT1 upregulation also increased Ca^+^_V_1.2 expression and calcium influx, promoting insulin secretion in β-cells 225. In addition, studies have shown that fucoglycan (FO) isolated from brown algae can ameliorate pancreatic β-cell injury and impaired insulin synthesis under diabetic conditions, and improve hyperglycemia, lower expression of SIRT1, PDX-1, and GLP-1R in the pancreas of diabetic mice. EX527 plays an important auxiliary role in this study, which can significantly reverse the beneficial effects of FO 226. Similarly, EX527 blocks the protective effect of curcumin on MIN6 (a mouse insulinoma cell line) cells exposed to HO 227. The above evidence suggests a significant protective effect of SIRT1 in diabetes mellitus.

## Conclusion and Perspectives

SIRT1, a protein deacetylase dependent on NAD^+^, has long been considered as an evolutionarily conserved life-regulating factor and is associated with many aging-related diseases 228. With the further study of SIRT1, another potential role of SIRT1 has been discovered -- the energy sensor of the body. SIRT1 is involved in hormone regulation, energy uptake, circadian rhythm, and metabolism. It also has potential therapeutic applications in cardiovascular disease, cancer and age-related diseases. The loss of SIRT1 leads to abnormal secretion of some hormones and metabolic disorders. SIRT1 regulates the function of pancreatic beta cells, improves insulin sensitivity and increases insulin secretion 11, 12. It has also been reported that Res, flavonoids and other compounds also affects insulin secretion by regulating the activity of SIRT1 in glucose-dependent insulin secretion 178, 229. These evidences suggest that SIRT1 is a key target of glucose metabolism and insulin resistance. In an orthotopic transplantation rat cholangiocarcinoma (CCA) model, the SIRT1 inhibitor sirtinol reduced tumor size and tumorigenic proteins (glioma-associated oncogene 1, phosphorylated extracellular signal-regulated kinase, and IL-6) expression 230. In addition, SIRT1 has been shown to promote progression of colorectal cancer 231. SIRT1 also plays a key role in gout/hyperuricemia, hypertension, hyperlipidaemia and other diseases. For example, SIRT1 regulate metabolist-related target molecules, including PPARγ, SREBP and LXRα, to improve the lipid metabolic environment. It has been reported that SIRT1 is closely related to adiponectin, leptin or resistance derived from adipose tissue, and the level of adiponectin is positively correlated with SIRT1, which can be used as an endocrine signal to mediate the browning of white adipose tissues 232. Myeloid-specific SIRT1 knockout increases hepatic steatosis and hypothalamic inflammation in mice fed a high-fat diet 233. In addition, SIRT1 is also involved in regulating the secretion of thyroid hormones 234, testosterone 235, aldosterone 236, estrogen 13, glucagon 198 cortisol and pituitary hormone 15. At the same time, a variety of small molecules compounds and natural products can act on SIRT1 to different degrees, and our current research has also confirmed that SIRT1 plays an important role in the development of endocrine and metabolic disorders. Notably, the above effects may be caused by different action mechanisms of SIRT1 streets, such as SIRT1/Keap1/Nrf2/HO-1 and PI3K/Akt/GSK-3β mediated oxidative stress, SIRT1/NF-κB mediated inflammatory response, SIRT1/PGC1α mediated mitochondrial damage, and SIRT1/FOXO mediated autophagy 237. These evidences provide a theoretical basis for SIRT1 as a therapeutic target for endocrine and metabolic diseases.

However, endocrine and metabolic regulation is a complex process, and the above two aspects interact with each other. Moreover, the treatment targeting SIRT1 is still in the preliminary stage. More basic research and more clinical trials are needed before patients can benefit from SIRT1-targeted therapies. Further research may focus on, 1) comprehensively expounding the complex regulatory mechanism of SIRT1 in endocrine and metabolic systems. 2) Exploring the degree of SIRT1's involvement in regulating energy homeostasis and its sensitivity to energy under physiological conditions. 3) Developing standards for assessing SIRT1 expression, especially SIRT1 content in blood and hormone, sugar and lipid metabolism levels, so as to predict endocrine and metabolic diseases. 4) Endocrine and metabolic diseases are closely related to daily diet. How can the body maintain appropriate SIRT1 levels to protect the body from lipid and glucose metabolism disorders? 5) Studying whether SIRT1 targeted therapy has suboptimal efficacy in clinical application. In conclusion, this review provides a comprehensive overview of SIRT1's role in endocrine and metabolic diseases and provides theoretical basis for SIRT1's potential as a novel therapeutic target.

## Figures and Tables

**Table 1:** The roles of SIRT1 agonists in endocrine and metabolic diseases.

Compound	Molecular formula	Molecular weight	Model	Vitro/Vivo	Dosage and Administration	Effects	Reference
Res	C_14_H_12_O_3_	228.24	Obesity rats models	Rats	AIN93G supplemented with 2 g/kg or 4 g/kg diet, 12 weeks	Res reduces adipocyte browning and fat loss, and improves other metabolic phenotypes, including hyperglycemia and hyperlipidemia in mice, which are mediated through SIRT1.	54
Res	C_14_H_12_O_3_	228.24	Yeast polysaccharide and potassium oxonate induced hyperuricemia model in C57BL/6 mice	C57BL/6 female mice	20 or 40 mg/kg, intragastrically, 0, 7, 14, 21 and 28day	SIRT1 and its activator, Res, have clear anti-hyperuricemia functions in this mouse model, which possible mechanism is the activation of ABCG2 in the ileum through the PGC-1α/PPARγ pathway.	91
Res	C_14_H_12_O_3_	228.24	Dehydroepiandrosterone-induced polycystic ovary syndrome rats	Rats	20 or 40 mg/kg, intraperitoneal injection, 28day	Res combined therapy may improve the weight gain, hormone profile, and ovarian follicular cell architecture by inducing antioxidant and anti-inflammatory systems via SIRT1 and AMPK activation in polycystic ovary syndrome.	126
Res	C_14_H_12_O_3_	228.24	Obesity model and nicotinamide-streptozotocin-high fat diet induced mild type 2 diabetes rats	Rats	60 ng/kg min, infusion into the lumen of the duodenum, 50 min	Res reverses a 3d high fat diet-induced reduction in duodenal-mucosal Sirt1 protein levels while also enhancing insulin sensitivity and lowering hepatic glucose production.	17
Res	C_14_H_12_O_3_	228.24	Particle-induced osteolysis (PIO) animal	C57BL/6 female mice	60 mg/kg, intragastrically, 2 weeks.	Res inhibits endoplasmic reticulum stress and reduces polyethylene particle - induced osteoclast differentiation and osteolysis.	144
SRT3025	C_31_H_31_N_5_O_2_S_2_	569.74	Apolipoprotein E-deficient mouse of atherosclerosis	C57BL/6J mice	3.18 g/kg diet, 12 weeks	SRT3025 can reduce plasma cholesterol levels, inflammation and atherosclerosis, and increase LDL receptor (Ldlr) and proprotein convertase subtilisin/kexin type 9 (Pcsk9) accumulation in the liver.	183
SRT3025	C_31_H_31_N_5_O_2_S_2_	569.74	Ovariectomy (OVX)-induced bone loss	C57BL/6J mice	50 and 100 mg/kg/day, Oral administration, 6 weeks.	Treatment with SRT3025 decreased bone sclerostin expression and increased cortical periosteal mineralizing surface and serum propeptide of type I procollagen, a bone formation marker.	197
SRT3025	C_31_H_31_N_5_O_2_S_2_	569.74	Streptozotocin (STZ)-induced diabetes	Male CD1 mice	3.18 g/kg, milled in chow, 20 weeks.	Treatment with SRT3025 diminished their glucagon secretion and proliferative activity in association with a reduction in the alpha cell associated transcription factor, Aristaless Related Homeobox (Arx)	198
SRT3025	C_31_H_31_N_5_O_2_S_2_	569.74	Advanced oxidation protein products (AOPPs) induce osteoporosis.	C57Bl/6 mice	50 mg/kg, in drinking water, 16 weeks.	SRT3025 inhibits the expression of sclerosclerin in bone cells by activating SIRT1, and reduced the resorptive activity and formation activity of bone tissue, thereby alleviating age-related bone loss	200
SRT3025	C_31_H_31_N_5_O_2_S_2_	569.74	Diabetes mouse	Old male db/db mouse	3.18 g/kg, milled in chow, 12 weeks.	While reducing hyperglycaemia and promoting beta cell expansion, enhancing the activity of SIRT1 facilitates a phenotypic change in a db/db mouse model of diabetes to one that more closely resembles the physiological state of torpor or hibernation.	238
Nicotinic acid	C_6_H_5_NO_2_	123.11	Hyperlipidemia and atherosclerosis mice	LDL receptor knockout mice	50, 250, or 1000 mg/kg, milled in chow, 8 weeks.	Nicotinic acid reduces total cholesterol, cholesterol esters, plasma triglycerides, atherosclerotic lesion size, lipid area, and aortic macrophage infiltration.	239
ginsenoside Rb2	C_53_H_90_O_22_	1079.27	Nonalcoholic fatty liver disease (NAFLD) and glucose tolerance model	C57BL/KsJ-Lepdb (db/db) mice	10 mg/kg/day, intraperitoneal injection, 4 weeks.	Rb2 alleviates hepatic lipid accumulation by restoring autophagy via the induction of sirt1 and activation of AMPK, and resultes in improved NAFLD and glucose tolerance.	174
Melatonin	C_13_H_16_N_2_O_2_	232.282	Apolipoprotein E-deficient mice (ApoE-/-)	C57BL/6 male mice and ApoE-/- male mice	10 mg/kg/day, dissolved in 1% ethanol and then diluted in tap water, 9 weeks	Melatonin treatment improves serum biochemical markers and hepatic morphological impairment and inhibited oxidative stress through its antioxidant properties and also by SIRT1 upregulation.	175
Melatonin	C_13_H_16_N_2_O_2_	232.282	Polycystic ovary syndrome (PCOS) and premature ovarian failure (POF) mice	ICR(CD-1) mice Liver Microsomes (Female)	15 mg/kg/day, intraperitoneal injection, 2 days	Melatonin suppresses FOXO1 via the PI3K-AKT axis, improves granulosa cell resistance to oxidative stress, and abolishes the autophagic response, from genes expression to the formation of autophagic vacuoles.	217
Melatonin	C_13_H_16_N_2_O_2_	232.282	chronic stress mice	Female BALB/c mice	20 mg/kg/day, intraperitoneal injection, 28 days	Melatonin alleviates chronic stress-induced oxidative meiosis defects in mouse MII oocytes by regulating SIRT1 and autophagy	61
SRT1720	C_25_H_24_ClN_7_OS	506.02236	Type 2 diabetic mice	Male C57BL/KsJ db/db mice	50 mg/kg/day, gavage,10 weeks.	SRT1720 inhibits the expression of HF1 α, GLUT1 and SNAIL by activating SIRT1, and alleviates and prevents diabetic induced renal fibrosis	211
SRT1720	C_25_H_24_ClN_7_OS	506.02236	High-fat diet mice	Adult female Kunming mice	50 mg/kg/day, intraperitoneal injection, 6 weeks.	SRT1720 may improve the follicle pool reserve in high-fat diet-induced obese female mice via activating SIRT1 signaling and suppressing mTOR signaling, thus extending the ovarian lifespan.	212
Metformin (MF)	C₄HN₅	129.164	polycystic ovary syndrome (PCOS) rats	Female rats	300 mg/kg/day, subcutaneous injection, 4 weeks.	MF can improve the reproductive and endocrine functions of rats with PCOS via the AMPKα-SIRT1 pathway.	128
Quercetin	CHO_7_	302.236	Letrozole-induced Polycystic ovary syndrome (PCOS) mice	Female Wistar rats	100 mg/kg/day, intragastric administration, 30 days.	Treatment with quercetin improves the PCOS related disturbances in estrous cycle, lipid profile, serum levels of testosterone, estradiol and progesterone, and insulin resistance. Besides, the expression levels of AMPK and SIRT-1 in ovarian tissue are upregulated in the rats which received quercetin. Quercetin also reverses the PCOS-induced alteration in adipose tissue levels of adiponectin, visfatin, and resistin.	129
